# One-Year Change in the H_2_FPEF Score After Catheter Ablation of Atrial Fibrillation in Patients With a Normal Left Ventricular Systolic Function

**DOI:** 10.3389/fcvm.2021.699364

**Published:** 2021-08-03

**Authors:** Min Kim, Hee Tae Yu, Tae-Hoon Kim, Jae-Sun Uhm, Boyoung Joung, Moon-Hyoung Lee, Hui-Nam Pak

**Affiliations:** ^1^Division of Cardiology, Chungbuk National University Hospital, Cheongju, South Korea; ^2^Division of Cardiology, Yonsei University Health System, Seoul, South Korea

**Keywords:** atrial fibrillation, catheter ablation, left venticular diastolic dysfunction, recurrent event, risk score

## Abstract

**Background:** It is unclear whether atrial fibrillation (AF) catheter ablation (AFCA) improves the left ventricular (LV) diastolic function. We evaluated the 1-year change in the H_2_FPEF score, which reflects the degree of LV diastolic function, after AFCA among patients with a normal LV systolic function.

**Methods and Results:** We included 1,471 patients (30.7% female, median age 60 years, paroxysmal-type AF 68.6%) who had available H_2_FPEF scores at baseline and at 1-year after AFCA to evaluate the 1-year change in the H_2_FPEF score (ΔH_2_FPEF score_[1−yr]_) after AFCA. Baseline high H_2_FPEF scores (≥6) were independently associated with the female sex, left atrium (LA) diameter, LV mass index, pericardial fat volume, and a low estimated glomerular filtration rate. One year after AFCA, decreased ΔH_2_FPEF scores_[1−yr]_ were associated with baseline H_2_FPEF scores of ≥6 [OR, 4.19 (95% CI, 2.88–6.11), *p* < 0.001], no diabetes [OR, 0.60 (95% CI, 0.37–0.98), *p* = 0.04], and lower pericardial fat volume [OR, 0.99 (95% CI, 0.99–1.00), *p* = 0.003]. Increased ΔH_2_FPEF scores_[1−yr]_ were associated with a baseline H_2_FPEF score of <6 [OR, 3.54 (95% CI, 2.08–6.04), *p* < 0.001] and sustained AF after a recurrence within 1 year [SustainAF_[1−yr]_; OR, 1.89 (95% CI, 1.01–3.54), *p* = 0.048]. Throughout a 56-month median follow-up, an increased ΔH_2_FPEF score_[1−yr]_ resulted in a poorer rhythm outcome of AFCA (at 1 year, log-rank *p* = 0.003; long-term, log-rank *p* = 0.010).

**Conclusions:** AFCA appears to improve LV diastolic dysfunction. However, SustainAF_[1−yr]_ may contribute to worsening LV diastolic dysfunction, and it was shown by increased ΔH_2_FPEF scores_[1−yr]_, which was independently associated with higher risk of AF recurrence rate after AFCA.

**Clinical Trial Registration:**ClinicalTrials.gov Identifier: NCT02138695.

## Introduction

Atrial fibrillation (AF) and underlying heart failure (HF) have been emerging topics of importance in the field of cardiovascular disease over the past 3 decades and frequently overlap (1, 2). Specifically, AF has been shown to follow HF with preserved ejection fraction (HFpEF) more frequently than HF with reduced ejection fraction (HFrEF) due to the differences in the left ventricular (LV) diastolic dysfunction and the left atrial (LA) remodeling process (2, 3). Prior studies have shown improvements in the LV systolic function (4), performance and quality of life (5), and mortality (6) after AF catheter ablation (AFCA) in HFrEF patients, suggesting that a reduction in AF may be sufficient for a clinical benefit. Nevertheless, there are no specific recommendations for the management of AF in HFpEF patients, and data regarding the efficacya of AFCA in patients with a normal LV systolic function and LV diastolic dysfunction are relatively limited. Although there have been a few studies reporting an improvement in the LV diastolic function after AFCA by maintaining sinus rhythm (7, 8), they adopted conventional approaches that used mainly symptoms and the LV ejection fraction (LVEF) for the diagnosis of HFpEF with various diagnostic accuracies (9). Recently, a novel scoring system has been developed, the H_2_FPEF score (10), which can estimate the probability of the underlying HFpEF through six clinical and echocardiographic characteristics, and can be feasibly applied in clinical practice. The aim of this study was to better understand the factors by which LV diastolic function worsens or improves after AF rhythm control by AFCA. In this study, we used the H_2_FPEF score at two time points, before and 1 year after the AFCA. We aimed to compare the cardiac structural and functional changes within a year and to evaluate the rhythm outcomes both within a year and over the long-term using the H_2_FPEF score.

## Materials and Methods

### Study Subjects

**Figure 1:**
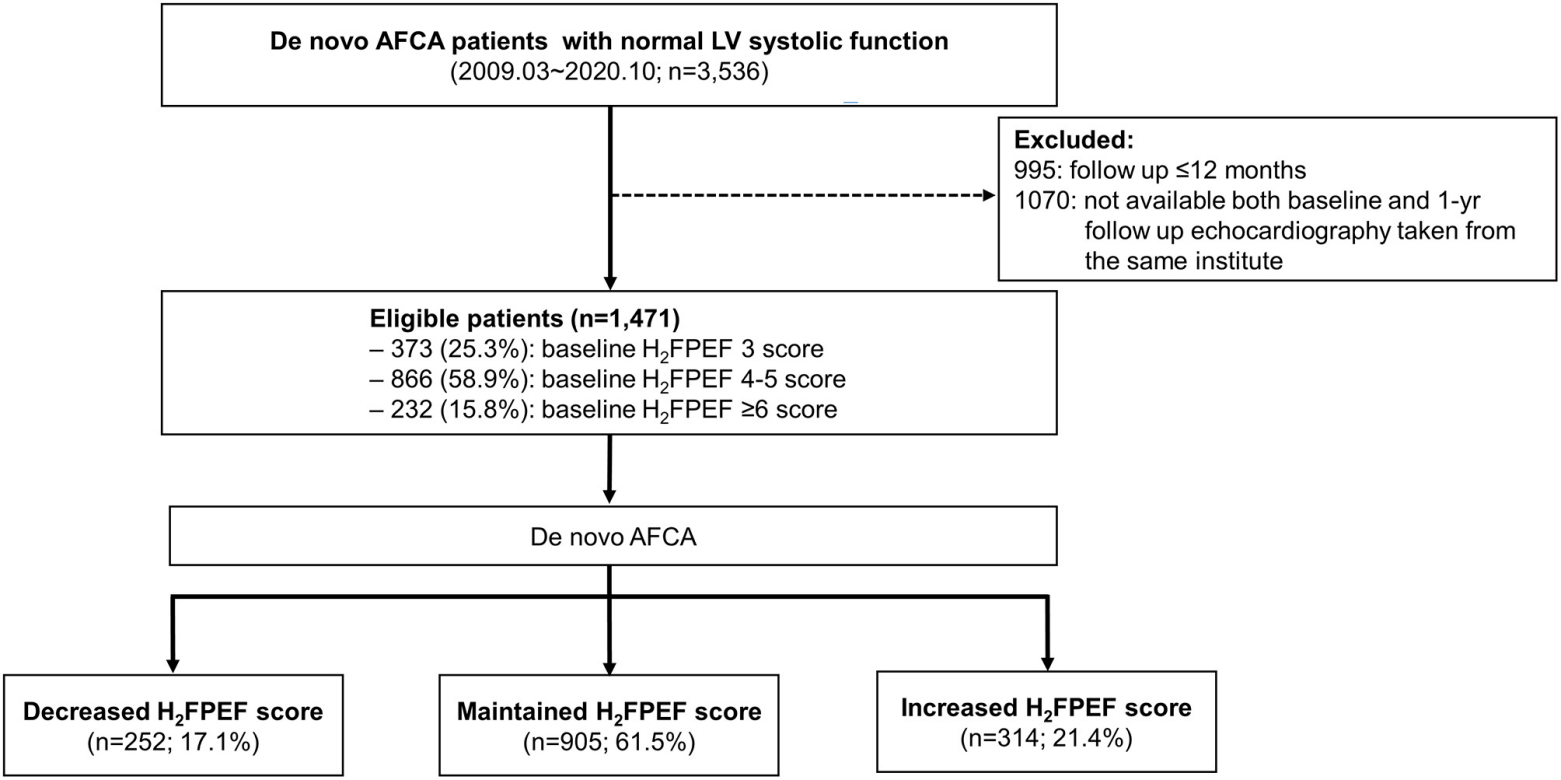
Screening and study flowchart of the patient selection. AF, atrial fibrillation; AFCA, atrial fibrillation catheter ablation; LV, left ventricular.

The study protocol adhered to the Declaration of Helsinki and was approved by the institutional review board of the Yonsei University Health system. All patients provided written informed consent for inclusion in the Yonsei AF Ablation Cohort Database (ClinicalTrials.gov Identifier: NCT02138695). From January 2009 to September 2019, 1,471 patients with a diagnosis of AF and a normal LVEF were identified as having clinical and echocardiographic information for the calculation of the H_2_FPEF score before AFCA and 1 year after AFCA. All patients underwent AFCA, and the indications for the AFCA complied with the latest guidelines (11). The exclusion criteria for the study were as follows: (1) a reduced LVEF, defined as <50%; (2) a follow-up duration <12 months; and (3) a repeat ablation within a year (Figure 1).

### Calculating the H_2_FPEF Score at Baseline and 1 Year After the Atrial Fibrillation Catheter Ablation

The H_2_FPEF score has six domains based on clinical and echocardiographic values: heaviness (body mass index >30 kg/m^2^, 2 points), hypertension (on two or more antihypertensive medicines, 1 point), atrial fibrillation (paroxysmal or persistent, 3 points), pulmonary hypertension (Doppler echocardiographic estimated pulmonary artery systolic pressure >35 mmHg, 1 point), elderly status (age >60 years, 1 point), and filling pressure (Doppler echocardiographic E/Em > 9, 1 point). The baseline H_2_FPEF scores were obtained within 3 months prior to the AFCA, and the 1-year H_2_FPEF scores were obtained 1 year after the AFCA with all clinical and echocardiographic values.

### Echocardiographic Measurement and Three-Dimensional Computed Tomography

Transthoracic echocardiography was conducted in all patients using commercially available devices (Vivid 7 or Vivid E9 from GE Healthcare, Chicago, IL, USA, or iE 33 from Philips, Amsterdam, the Netherlands) as recommended by the American Society of Echocardiography (12). Standard images were obtained in the parasternal and apical views through two-dimensional (2D), Doppler, and M-mode imaging, including the LA anteroposterior diameter and the LV end-systolic and end-diastolic dimensions (LVESD and LVEDD). The early Doppler mitral inflow (E) was recorded using pulsed waves from the apical window, with a 1- to 3-mm pulsed Doppler sample volume placed between the tips and mitral leaflets during diastole. The early diastolic mitral annular velocity (Em) was recorded as the peak early diastolic tissue velocity using color Doppler tissue imaging of the septal mitral annulus. The ratio of the early diastolic mitral inflow velocity to the early diastolic mitral annular velocity (E/Em) was calculated. Tricuspid regurgitation (TR) and estimated right atrial (RA) pressure were evaluated using the recommended methods, and the right ventricular systolic pressure (RVSP) was calculated as 4 × (TR jet)^2^ + estimated RA pressure (13). The initial and 1 year after AFCA, the echocardiographies used to estimate the H_2_FPEF scores were those performed during an elective visit on stable medication.

Three-dimensional spiral computed tomography (CT) (64-channel, Light Speed Volume CT from GE Healthcare, Chicago, IL, USA, or Brilliance 63 from Philips, Amsterdam, the Netherlands) was performed in all patients, and the scans were analyzed using an imaging-processing workstation (Aquarius; TeraRecon, Inc., Foster City, CA, USA). The LA volume and pericardial fat volume measurements have been described in previous studies (14, 15).

### Electrophysiologic Characterization and Radiofrequency Catheter Ablation

Intracardiac electrograms were obtained using the Prucka CardioLab™ Electrophysiology system (GE Healthcare, Chicago, IL, USA). A 3D electro-anatomical map (Ensite NavX; Abbott Laboratories, Chicago, IL, USA; CARTO3; Johnson & Johnson Inc., USA) was generated using a circumferential pulmonary vein-mapping catheter through a long sheath (Schwartz left 1; Abbott Laboratories, Chicago, IL, USA) and by merging the 3D geometry generated by the electroanatomic mapping system with the corresponding 3D spiral CT images. Left atrium electrogram voltage maps were generated during high right atrial pacing at 500 ms to prevent rate-dependent activation changes and by measuring mean peak-to-peak voltage as previously described (16). All patients underwent a *de novo* procedure with a circumferential pulmonary vein isolation (CPVI). The endpoint of the CPVI was the electric isolation of the PV potentials and bidirectional block of the PVs. We tested whether there was an immediate recurrence of AF within 10 min after cardioversion with an isoproterenol infusion (5–20 μg/min depending on the ß-blocker used with a target sinus heart rate of 120 bpm) to find extra-PV foci triggers, then confirmed successful CPVI 30 min after the initial isolation. Extra-PV foci triggers under an isoproterenol infusion were ablated as much as possible if they were consistent and reproducible. Then, we ended the *de novo* procedure. The detailed procedural techniques and strategies for the AFCA have been presented in our previous studies (17, 18).

### Post-ablation Management and Rhythm Follow-Up

The patients were discharged without any antiarrhythmic drugs (AADs) with the exception of those who had symptomatic frequent atrial premature beats, non-sustained atrial tachycardia (AT), early recurrence of AF on telemetry during the admission period, or recurrent extra-PV foci triggers after the AFCA procedure (13.7%). The clinical and cardiac rhythm information was obtained regularly from an outpatient clinic at 1, 3, 6, and 12 months, and every 6 months thereafter (or whenever symptoms developed). All patients underwent electrocardiogram recordings at every visit, and 24-h Holter monitoring was performed at 3 and 6 months, and every 6 months thereafter, according to the latest guidelines (11). AF recurrence was defined as any episode of AF or AT of at least 30 s in duration. Any ECG documentation of an AF recurrence within the 3-month blanking period was diagnosed as an early recurrence, and an AF recurrence of more than 3 months after the AFCA was diagnosed as a clinical recurrence. We evaluated the time point of the clinical recurrence as follows: within 1 year as a short-term and beyond 1 year as a long-term recurrence. We also estimated the quality of the AF control after the AFCA. We defined patients with sustained AF/AT as those who remained in a sustaining AF/AT rhythm (>30 s) on the final follow-up after the AFCA despite AADs or electrical cardioversion.

### Statistical Analysis

The baseline characteristics of the patients were compared using descriptive statistics and presented as median (interquartile interval) values for continuous variables and as numbers (percentages) for categorical variables. To compare the baseline characteristics according to the baseline H_2_FPEF and the 1-year change in the H_2_FPEF score (ΔH_2_FPEF score_[1−yr]_) categories, the Mantel–Haenszel chi-squared test was used for categorical variables, and the Kruskal–Wallis H test was used for continuous variables. To identify the factors associated with the baseline H_2_FPEF and ΔH_2_FPEF score_[1−yr]_, univariate and multivariable logistic regression analyses were performed. Multivariable Cox proportional hazard analyses were performed to evaluate the association of the baseline H_2_FPEF and ΔH_2_FPEF score_[1−yr]_ with a clinical recurrence in both the short- and long-term periods. A multivariable regression analysis included those variables with significant *p*-values of < 0.1 in the univariate analysis. The Cox proportional hazards assumption was tested based on Schoenfeld residuals. Two-sided *p*-values < 0.05 were considered statistically significant. The statistical analyses were conducted using SAS version 9.4 (SAS Institute) and R version 4.0.0 (R Foundation for Statistical Computing) software.

## Results

### Characteristics of the Patients With High H_2_FPEF Scores

Table 1 summarizes patient characteristics depending on the H_2_FPEF score. In the 1,471 patients, the median (IQR) age was 60 (53, 68) years, 30.7% were female, and 68.6% had paroxysmal-type AF. The baseline H_2_FPEF scores were 3 points in 373 patients (25.3%), 4–5 points in 866 (58.9%), and ≥6 points in 232 (15.8%) patients. Patients with higher H_2_FPEF scores were older and had a higher body mass index, hypertension, estimated RVSP, and higher E/Em, as those variables that composed this score. In the higher H_2_FPEF score-group, the CHA_2_DS_2_-VASc score (*p* < 0.001), prevalence of diabetes (*p* < 0.001), a prior stroke (p = 0.004), vascular disease (*p* < 0.001), or hypertrophic cardiomyopathy (*p* = 0.007) were higher. The CT-measured LA volume (*p* < 0.001), pericardial fat volume (*p* < 0.001), and LA peak pressure (*p* = 0.004) were higher, and the endocardial bipolar LA voltage (*p* < 0.001) and eGFR (*p* < 0.001) were significantly lower in the higher H_2_FPEF score-group. In the multivariate logistic regression analysis (Supplementary Table 1), high baseline H_2_FPEF scores (≥6) were independently associated with the female sex [OR, 2.31 (1.35–3.93), *p* = 0.002], higher left atrial (LA) diameter [OR, 1.09 (1.04–1.13), *p* < 0.001], LV mass index [OR 1.02 (1.01–1.03), *p* = 0.002], pericardial fat volume [OR, 1.01 (1.00–1.01), *p* = 0.015], and lower eGFR [OR, 0.98 (0.97–0.99), *p* < 0.001].

**Table 1:** Baseline characteristics according to the baseline H_2_FPEF score stratification in atrial fibrillation (AF) patients with a normal left ventricular ejection fraction (LVEF).

	**Overall (*N* = 1,471)**	**3 score (*N* = 373)**	**4–5 score (*N* = 866)**	**≥6 score (*N* = 232)**	***p*-value**
Age, years	60 (53, 68)	52 (46, 56)	63 (56, 69)	68 (63, 74)	<0.001
Age over 65 years, *n* (%)	463 (31.5)	0 (0)	316 (36.5)	147 (63.4)	<0.001
Female, *n* (%)	452 (30.7)	65 (17.4)	282 (32.6)	105 (45.3)	<0.001
FU duration, months	56 (32, 87)	54 (31, 85)	58 (33, 89)	57 (33, 86)	0.232
BMI, kg/m^2^	24.6 (23.0, 26.6)	24.5 (23.0, 26.4)	24.4 (22.8, 26.4)	25.4 (23.6, 28.0)	<0.001
Paroxysmal AF, *n* (%)	1,009 (68.6)	266 (71.3)	597 (68.9)	146 (62.9)	0.092
Smoking, *n* (%)					0.014
Never	957 (65.1)	232 (62.2)	555 (64.1)	170 (73.3)	
Former/current	514 (34.9)	141 (37.8)	311 (35.9)	62 (26.7)	
Alcohol, *n* (%)					<0.001
Never	771 (52.4)	159 (42.6)	461 (53.2)	151 (65.1)	
Former/current	700 (47.6)	214 (57.4)	405 (46.8)	81 (34.9)	
CHA_2_DS_2_-VASc score	2 (1, 3)	0 (0, 1)	2 (1, 3)	3 (2, 4)	<0.001
Heart failure, *n* (%)^*^	96 (6.5)	14 (3.8)	51 (5.9)	31 (13.4)	<0.001
Hypertension, *n* (%)	714 (48.5)	54 (14.5)	450 (52.0)	210 (90.5)	<0.001
Diabetes, *n* (%)	222 (15.1)	27 (7.2)	134 (15.5)	61 (26.3)	<0.001
Prior stroke/TIA, *n* (%)	180 (12.2)	25 (6.7)	113 (13.0)	42 (18.1)	0.004
Vascular disease, *n* (%)	194 (13.2)	10 (2.7)	135 (15.6)	49 (21.1)	<0.001
Hypertrophic cardiomyopathy, *n* (%)	36 (2.4)	1 (0.3)	28 (3.2)	7 (3.0)	0.007
Obstructive sleep apnea, *n* (%)	16 (1.1)	3 (0.8)	11 (1.3)	2 (0.9)	0.720
Chronic obstructive pulmonary disease, *n* (%)	21 (1.4)	2 (0.5)	14 (1.6)	5 (2.2)	0.202
Thyroid disease, *n* (%)	108 (7.3)	20 (5.4)	67 (7.7)	21 (9.1)	0.188
**Laboratory**					
eGFR, ml/min/1.73 m^2^	80.3 (69.0, 92.9)	86.1 (75.3, 97.2)	79.5 (68.9, 92.1)	72.0 (59.4, 84.3)	<0.001
hs-CRP, mg/dl	0.7 (0.5, 1.5)	0.7 (0.3, 1.3)	0.8 (0.5, 1.4)	1.0 (0.6, 2.1)	<0.001
**Echocardiography**					
LA diameter, mm	41 (37, 45)	39 (36, 42)	41 (37, 45)	44 (41, 48)	<0.001
LAVI, ml/m^2^	35.0 (28.3, 43.0)	30.3 (25.8, 35.9)	35.6 (28.7, 43.4)	42.0 (35.0, 50.5)	<0.001
LVEF, %	65 (60, 69)	63 (60, 68)	65 (61, 69)	65 (61, 70)	0.009
E/Em	9.2 (7.8, 12.0)	7.0 (6.0, 8.0)	10.0 (8.0, 12.0)	12.9 (11.0, 15.4)	<0.001
E, m/s	0.7 (0.6, 0.8)	0.7 (0.6, 0.8)	0.7 (0.6, 0.8)	0.8 (0.6, 1.0)	<0.001
Em, cm/s	7.0 (6.0, 9.0)	9.5 (8.0, 11.0)	7.0 (5.6, 8.7)	6.0 (5.0, 7.2)	<0.001
TR jet, m/s	2.3 (2.1, 2.5)	2.1 (2.0, 2.3)	2.3 (2.1, 2.5)	2.5 (2.3, 2.8)	<0.001
RVSP, mmHg	26 (22, 30)	24 (21, 27)	26 (23, 30)	30 (26, 37)	<0.001
LVEDD, mm	50 (46, 53)	50 (47, 52)	50 (46, 52)	50 (46, 53)	0.412
LVESD, mm	33 (30, 36)	33 (31, 36)	33 (30, 35)	33 (30, 36)	0.140
LVMI, g/m^2^	90.9 (79.4, 102.9)	85.0 (75.8, 94.8)	91.3 (80.1, 104.4)	99.2 (87.9, 112.6)	<0.001
**3D-CT, ml**					
Pericardial fat volume	101.1 (70.8, 140.9)	91.2 (63.8, 135.3)	101.9 (70.9, 143.3)	113.8 (81.5, 156.1)	<0.001
LA volume	147.3 (120.8, 178.2)	132.3 (113.4, 163.2)	149.0 (122.6, 177.9)	163.9 (140.4, 195.1)	<0.001
**Voltage, mV**					
LA mean voltage	1.3 (0.8, 1.8)	1.5 (1.0, 2.0)	1.3 (0.8, 1.7)	1.1 (0.7, 1.5)	<0.001
**LA pressure, mmHg**					
Peak	20 (15, 27)	20 (15, 25)	20 (15, 27)	22 (17, 29)	0.004
Nadir	4 (1, 8)	4 (1, 8)	5 (1, 8)	5 (1, 9)	0.348
Mean	11 (8, 16)	11 (7, 15)	11 (8, 16)	13 (9, 17)	0.006
**Medication**, ***n*****(%)**					
ACEi/ARB	493 (33.5)	33 (8.8)	293 (33.9)	167 (72.0)	<0.001
Beta-blocker	487 (33.1)	104 (27.9)	274 (31.7)	109 (47.0)	<0.001
Statin	491 (33.4)	57 (15.3)	322 (37.2)	112 (48.3)	<0.001
AAD^†^	200 (13.7)	40 (10.8)	118 (13.7)	42 (18.1)	0.038
**Recurrence after AFCA**, ***n*****(%)**					
Early recurrence	448 (30.5)	111 (29.8)	271 (31.3)	66 (28.4)	0.666
**Clinical recurrence**					
1-year duration	257 (17.5)	62 (16.6)	152 (17.6)	43 (18.5)	0.830
Total duration	611 (41.5)	145 (38.9)	358 (41.3)	108 (46.6)	0.173
**Sustained AF**					
1-year duration	56 (3.8)	16 (4.3)	28 (3.2)	12 (5.2)	0.333
Total duration	95 (6.5)	27 (7.2)	51 (5.9)	17 (7.3)	0.568

*The data are presented as the number (%) and median (interquartile interval). Non-parametric continuous variables as assessed by the Kolmogorov–Smirnov method were analyzed by the Mann–Whitney U-test. AAD, antiarrhythmic drug; ACEi, angiotensin-converting-enzyme inhibitor; AF, atrial fibrillation; AFCA, atrial fibrillation catheter ablation; ARB, angiotensin receptor blocker; BMI, body mass index; E/Em, ratio of the early diastolic mitral inflow velocity (E) to the early diastolic mitral annular velocity (Em); FU, follow up; eGFR, estimated glomerular filtration rate; hs-CRP, high sensitivity C-reactive protein; LA, left atrium; LVEDD, left ventricular end diastolic dimension; LVEF, left ventricular ejection fraction; LVESD, left ventricular end systolic dimension; LVMI, left ventricular mass index; RVSP, right ventricular systolic pressure; TIA, transient ischemic attack; TR, tricuspid regurgitation*.

* *Defined as conventional HFpEF diagnosis riteria: left ventricular ejection fraction ≥50% with exertional dyspnea that was not caused by extracardiac causes*.

† *Including class Ic and III drugs, taken after catheter ablation*.

### ΔH_2_FPEF Score_[1-yr]_

**Figure 2:**
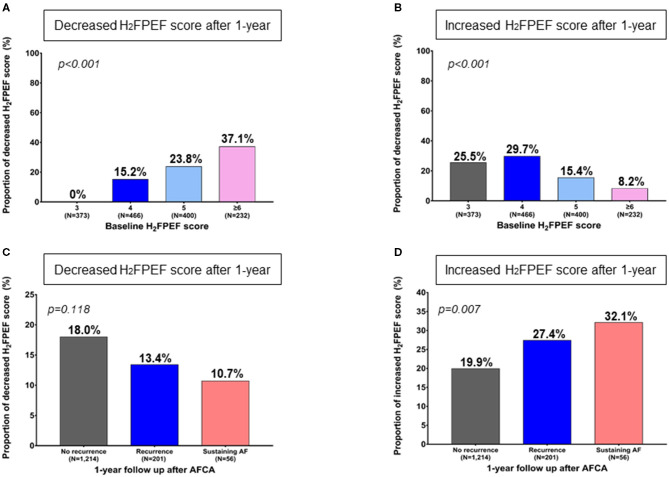
Change in the 1-year H_2_FPEF scores after the AFCA, according to the baseline H_2_FPEF scores **(A, B)** and rhythm outcomes within a year **C, D)**. AF, atrial fibrillation; AFCA, atrial fibrillation catheter ablation.

One year after the AFCA, the H_2_FPEF scores decreased in 17.1% of the patients (252), were maintained in 61.5% (905), and increased in 21.4% (314) (Table 2). A reduction in the ΔH_2_FPEF score_[1−yr]_ was more commonly observed in patients with high baseline H_2_FPEF scores (Figure 2A) and was independently associated with baseline H_2_FPEF scores of ≥6 [OR, 4.19 (2.88–6.11), *p* < 0.001], the absence of diabetes [OR, 0.60 (0.37–0.98), *p* = 0.04], a higher LVEF [OR, 1.03 (1.01–1.06), *p* = 0.011], and a lower pericardial fat volume [OR, 0.99 (0.99–1.00), *p* = 0.003; Table 3]. Increased ΔH_2_FPEF scores_[1−yr]_ were less commonly observed in patients with high H_2_FPEF scores (Figure 2B) and were associated with a baseline H_2_FPEF score of <6 [OR, 3.54 (2.08–6.04), *p* < 0.001], sustained AF after a recurrence within a year [SustainAF_[1−yr]_; OR, 1.89 (1.01–3.54), *p* = 0.048], the LA volume [OR, 1.00 (1.00–1.01), *p* = 0.029], and the pericardial fat volume [OR, 1.00 (1.00–1.01), *p* = 0.032; Table 4].

**Table 2:** Baseline characteristics according to the change in the H_2_FPEF scores in AF patients with a normal LVEF, 1-year after the atrial fibrillation catheter ablation (AFCA).

	**Decreased (−2, −1) (*N* = 252)**	**Maintained (0) (*N* = 905)**	**Increased (+1, +2, +3) (*N* = 314)**	***p*-value**
Age, years^*^	62 (55, 68)	59 (52, 67)	60 (58, 68)	<0.001
Age over 65 years, *n* (%)	82 (32.5)	272 (30.1)	109 (34.7)	0.286
v Female, *n* (%)	78 (31.0)	283 (31.3)	91 (29.0)	0.748
FU duration, months	53 (31, 88)	58 (32, 87)	58 (33, 89)	0.467
BMI, kg/m^2^	24.4 (22.8, 26.1)	24.6 (23.0, 26.7)	24.6 (23.2, 26.7)	0.445
Paroxysmal AF, *n* (%)	183 (72.6)	622 (68.7)	204 (65.0)	0.148
Smoking, *n* (%)				0.152
Never	157 (62.3)	606 (67.0)	194 (61.8)	
Former/current, *n* (%)	95 (37.7)	299 (33.0)	120 (38.2)	
Alcohol				0.222
Never	141 (56.0)	477 (52.7)	153 (48.7)	
Former/current	111 (44.0)	428 (47.3)	161 (51.3)	
CHA_2_DS_2_-VASc score	2 (1, 3)	1 (0, 3)	2 (1, 3)	0.238
Heart failure, *n* (%)^†^	20 (7.9)	56 (6.2)	20 (6.4)	0.606
Hypertension, *n* (%)	133 (52.8)	431 (47.6)	150 (47.8)	0.335
Diabetes, *n* (%)	29 (11.5)	142 (15.7)	51 (16.2)	0.212
Prior stroke/TIA, *n* (%)	37 (14.7)	108 (11.9)	35 (11.1)	0.401
Vascular disease, *n* (%)	33 (13.1)	115 (12.7)	46 (14.6)	0.680
Hypertrophic cardiomyopathy, *n* (%)	11 (4.4%)	17 (1.9%)	8 (2.5%)	0.077
Obstructive sleep apnea, *n* (%)	2 (0.8)	12 (1.3)	2 (0.6)	0.529
Chronic obstructive pulmonary disease, *n* (%)	3 (1.2)	12 (1.3)	6 (1.9)	0.709
Thyroid disease, *n* (%)	16 (6.3)	68 (7.5)	24 (7.6)	0.800
**Laboratory**				
eGFR, ml/min/1.73m^2^	80.3 (69.8, 91.4)	81.0 (69.4, 93.8)	79.0 (66.7, 90.7)	0.265
hs-CRP, mg/dl	0.8 (0.5, 1.4)	0.7 (0.5, 1.5)	0.8 (0.6, 1.8)	0.170
**Echocardiography**				
LA diameter, mm	41 (38, 45)	41 (37, 45)	41 (37, 46)	0.264
LAVI, ml/m^2^	36.2 (29.4, 44.3)	34.5 (27.7, 42.7)	35.5 (28.4, 43.6)	0.134
LVEF, %	66 (62, 70)	64 (60, 69)	65 (60, 69)	0.005
E/Em,	11.0 (10.0, 13.0)	9.0 (7.0, 12.0)	8.4 (7.3, 9.0)	<0.001
E, m/s	0.8 (0.6, 0.9)	0.7 (0.6, 0.8)	0.7 (0.6, 0.8)	<0.001
Em, cm/s	6.3 (5.0, 8.0)	7.5 (6.0, 9.3)	8.0 (6.0, 9.0)	<0.001
TR jet, m/s	2.4 (2.1, 2.7)	2.2 (2.1, 2.4)	2.3 (2.1, 2.4)	<0.001
RVSP, mmHg	28 (23, 36)	25 (22, 29)	26 (22, 29)	<0.001
LVEDD, mm	50 (46, 53)	50 (47, 53)	49 (46, 52)	0.309
LVESD, mm	33 (30, 36)	33 (30, 36)	33 (31, 35)	0.285
LVMI, g/m^2^	92.9 (80.5, 107.1)	90.1 (79.4, 102.3)	91.6 (79.2, 102.3)	0.223
**3D-CT, ml**
Pericardial fat volume	96.8 (63.8, 134.1)	100.9 (71.0, 140.6)	108.4 (78.3, 150.3)	0.012
LA volume	144.0 (121.2, 174.2)	146.9 (119.8, 176.6)	152.7 (124.2, 184.1)	0.084
**Voltage, mV**				
LA mean voltage	1.4 (0.9, 1.8)	1.3 (0.9, 1.8)	1.3 (0.8, 1.7)	0.277
**LA pressure, mmHg**				
Peak	20 (15, 27)	20 (15, 27)	21 (16, 27)	0.394
Nadir	5 (1, 9)	4 (1, 8)	5 (1, 8)	0.681
Mean	11 (8, 16)	11 (8, 16)	12 (8, 16)	0.333
**1-year change in the Echocardiography**, **Δ**				
**Δ** LA diameter, mm	−4 (−6, −1)	−3 (−5, 0)	−2 (−5, 1)	<0.001
**Δ** LAVI, ml/m^2^	−8.0 (−14.0, −2.3)	−5.2 (−10.7, −0.2)	−3.6 (−9.1, 1.9)	<0.001
**Δ** LVEF, %	1 (−4, 5)	1 (−3, 6)	1 (−3, 5)	0.459
**Δ** E/Em	−3.0 (−4.2, −1.4)	0 (−1.1, 1.5)	2.8 (1.0, 4.4)	<0.001
**Δ** E, m/s	−0.1 (−0.2, 0)	0 (−0.2, 0)	0 (−0.1, 0.1)	<0.001
**Δ** Em, cm/s	0.9 (-1.0, 2.0)	−0.8 (−2.0, 0.5)	−1.4 (-3.0, 0)	<0.001
**Δ** TR jet, m/s	−0.1 (−0.4, 0)	0 (−0.2, 0.2)	0.1 (−0.1, 0.4)	<0.001
**Δ** RVSP, mmHg	−3 (−9, 0)	−1 (−4, 3)	2.2 (−3, 8)	<0.001
**Δ** LVEDD, mm	−1 (−3, 1)	0 (−2, 2)	0 (−2, 2)	<0.001
**Δ** LVESD, mm	−1 (−3, 1)	−1 (−3, 1)	0 (−2, 2)	0.007
**Δ** LVMI, g/m^2^	−2.8 (−13.9, 5.6)	1.0 (−9.4, 9.4)	3.3 (−8.8, 13.1)	<0.001
**Medication**, ***n*****(%)**				
ACEi/ARB	96 (38.1)	298 (33.0)	99 (31.5)	0.218
Beta-blocker	93 (36.9)	285 (31.5)	109 (34.7)	0.220
Statin	96 (38.1)	289 (32.0)	106 (33.8)	0.188
AAD^‡^	30 (12.0)	118 (13.1)	52 (16.6)	0.204
**Recurrence after the AFCA**, ***n*****(%)**				
Early recurrence	68 (27.0)	281 (31.0)	99 (31.5)	0.416
**Clinical recurrence**				
1-year duration	33 (13.1)	151 (16.7)	73 (23.2)	0.004
Total duration	99 (39.3)	361 (39.9)	151 (48.1)	0.029
**Sustained AF**				
1-year duration	6 (2.4)	32 (3.5)	18 (5.7)	0.093
Total duration	14 (5.6)	59 (6.5)	22 (7.0)	0.774

*The data are presented as the number (%) and median (interquartile interval). Non-parametric continuous variables as assessed by the Kolmogorov–Smirnov method, were analyzed by the Mann–Whitney U-test. AAD, antiarrhythmic drug; ACEi, angiotensin-converting-enzyme inhibitor; AF, atrial fibrillation; AFCA, atrial fibrillation catheter ablation; ARB, angiotensin receptor blocker; BMI, body mass index; EEm, ratio of the early diastolic mitral inflow velocity (E) to the early diastolic mitral annular velocity (Em); FU, follow up; eGFR, estimated glomerular filtration rate; hs-CRP, high sensitivity C-reactive protein; LA, center atrium; LVEDD, center ventricular end diastolic dimension; LVEF, center ventricular ejection fraction; LVESD, center ventricular end systolic dimension; LVMI, center ventricular mass index; RVSP, right ventricular systolic pressure; TIA, transient ischemic attack; TR, tricuspid regurgitation*.

* *Increased vs. maintained, p < 0.001; increased vs. decreased, p = 0.263; maintained vs. decreased, p = 0.105*.

† *Defined as conventional HFpEF diagnosis criteria: center ventricular ejection fraction ≥50% with exertional dyspnea that was not caused by extracardiac causes*.

‡ *Including class Ic and III drugs, taken after catheter ablation*.

**Table 3:** Logistic regression analysis for predictors of decreased H_2_FPEF scores, 1-year after the AFCA.

	**Univariate analysis**		**Multivariable analysis**	
	**Unadjusted HR** ** (95% CI)**	***p*-value**	**Adjusted HR** ** (95% CI)**	***p*-value**
**Baseline H**_**2**_ **FPEF scores**				
<6 score (reference)	1.00 (reference)		1.00 (reference)	
≥6 score	3.81 (2.79–5.21)	<0.001	4.19 (2.88–6.11)	<0.001
Age	1.01 (0.99–1.02)	0.285		
Female	1.01 (0.76–1.36)	0.932		
Paroxysmal AF	1.26 (0.93–1.70)	0.135		
Body mass index	0.98 (0.94–1.03)	0.484		
Smoking	1.16 (0.87–1.53)	0.314		
Alcohol	0.84 (0.64–1.11)	0.217		
Heart failure^*^	1.30 (0.78–2.16)	0.321		
Hypertension	1.23 (0.94–1.61)	0.139		
Diabetes	0.69 (0.46–1.05)	0.082	0.60 (0.37–0.98)	0.040
Prior stroke/TIA	1.29 (0.88–1.91)	0.194		
Vascular disease	0.99 (0.66–1.48)	0.962		
CHA_2_DS_2_-VASc score	1.05 (0.96–1.14)	0.306		
LA diameter	1.00 (0.98–1.03)	0.708		
LVEF	1.04 (1.01–1.06)	0.002	1.03 (1.01–1.06)	0.011
E/Em	1.13 (1.09–1.66)	<0.001		
TR jet velocity	1.16 (0.98–1.36)	0.077		
RVSP	1.09 (1.06–1.11)	<0.001		
LVEDD	1.01 (0.98–1.05)	0.412		
LVESD	0.97 (0.94–1.01)	0.145		
LVMI	1.01 (1.00–1.01)	0.062	1.00 (0.99–1.01)	0.708
eGFR	1.00 (0.99–1.01)	0.745		
hs-CRP	1.00 (0.99–1.00)	0.665		
Pericardial fat volume	1.00 (1.00–1.00)	0.087	0.99 (0.99–1.00)	0.003
LA volume	1.00 (0.99–1.01)	0.540		
LA mean voltage	1.03 (0.81–1.30)	0.825		
LA peak pressure	0.99 (0.97–1.01)	0.166		
**Sustained AF** ^†^				
1-year duration	0.57 (0.24–1.35)	0.199		
Total duration	0.83 (0.46–1.48)	0.522		

*AF, atrial fibrillation; AFCA, atrial fibrillation catheter ablation; BMI, body mass index; EEm, ratio of the early diastolic mitral inflow velocity (E) to the early diastolic mitral annular velocity (Em); eGFR, estimated glomerular filtration rate; hs-CRP, high sensitivity C-reactive protein; LA, left atrium; LVEDD, left ventricular end diastolic dimension; LVEF, left ventricular ejection fraction; LVESD, left ventricular end systolic dimension; LVMI, left ventricular mass index; RVSP, right ventricular systolic pressure; TIA, transient ischemic attack; TR, tricuspid regurgitation*.

* *Defined as conventional HFpEF diagnosis criteria: left ventricular ejection fraction ≥50% with exertional dyspnea that was not caused by extracardiac causes*.

† *Defined as patients who remained in a sustained AF rhythm (>30 s) on the final follow-up date despite antiarrhythmic drugs or electrical cardioversion*.

**Table 4:** Logistic regression analysis for predictors of increased H_2_FPEF scores, 1-year after the AFCA.

	**Univariate analysis**		**Multivariable analysis**	
	**Unadjusted OR** ** (95% CI)**	***p*-value**	**Adjusted OR** ** (95% CI)**	***p*-value**
**Baseline H**_**2**_ **FPEF scores**
<6 score	1.14 (0.89–1.46)	0.311	3.54 (2.08–6.04)	<0.001
≥6 score (reference)	1.00 (reference)		1.00 (reference)	
Age	1.03 (1.01–1.04)	<0.001		
Female	0.90 (0.68–1.18)	0.450		
Paroxysmal AF	0.81 (0.62–1.05)	0.114		
Body mass index	1.02 (0.98–1.07)	0.323		
Smoking	1.20 (0.93–1.55)	0.170		
Alcohol	1.21 (0.94–1.55)	0.140		
CHA_2_DS_2_-VASc score	1.00 (0.92–1.08)	0.965		
Heart failure^*^	0.97 (0.58–1.61)	0.899		
Hypertension	0.96 (0.75–1.23)	0.759		
Diabetes	1.12 (0.80–1.57)	0.521		
Prior stroke/TIA	0.88 (0.59–1.30)	0.507		
Vascular disease	1.17 (0.82–1.67)	0.389		
LA diameter	1.02 (0.99–1.04)	0.168		
LVEF	1.00 (0.98–1.02)	0.813		
E/Em	0.89 (0.86–0.93)	<0.001		
TR jet velocity	0.81 (0.56–1.18)	0.276		
RVSP	0.98 (0.96–1.00)	0.071		
LVEDD	0.98 (0.95–1.01)	0.271		
LVESD	0.99 (0.96–1.02)	0.484		
LVMI	1.00 (0.99–1.01)	0.595		
eGFR	1.00 (0.99–1.00)	0.122		
hs-CRP	0.99 (0.97–1.02)	0.435		
Pericardial fat volume	1.00 (1.00–1.01)	0.012	1.00 (1.00–1.01)	0.032
LA volume	1.00 (1.00–1.01)	0.023	1.00 (1.00–1.01)	0.029
LA mean voltage	0.84 (0.67–1.05)	0.126		
LA peak pressure	1.01 (0.99–1.02)	0.405		
**Sustained AF** ^†^
1-year duration	1.79 (1.01–3.18)	0.047	1.89 (1.01–3.54)	0.048
Total duration	1.12 (0.68–1.83)	0.656		

*AF, atrial fibrillation; AFCA, atrial fibrillation catheter ablation; BMI, body mass index; EEm, ratio of the early diastolic mitral inflow velocity (E) to the early diastolic mitral annular velocity (Em); eGFR, estimated glomerular filtration rate; hs-CRP, high sensitivity C-reactive protein; LA, left atrium; LVEDD, left ventricular end diastolic dimension; LVEF, left ventricular ejection fraction; LVESD, left ventricular end systolic dimension; LVMI, left ventricular mass index; RVSP, right ventricular systolic pressure; TIA, transient ischemic attack; TR, tricuspid regurgitation*.

* *Defined as conventional HFpEF diagnosis criteria: left ventricular ejection fraction ≥50% with exertional dyspnea that was not caused by extracardiac causes*.

† *Defined as patients who remained in a sustained AF rhythm (>30 sec) on the final follow-up date despite antiarrhythmic drugs or electrical cardioversion*.

### Rhythm Outcomes After Atrial Fibrillation Catheter Ablation and the H_2_FPEF Score

**Figure 3:**
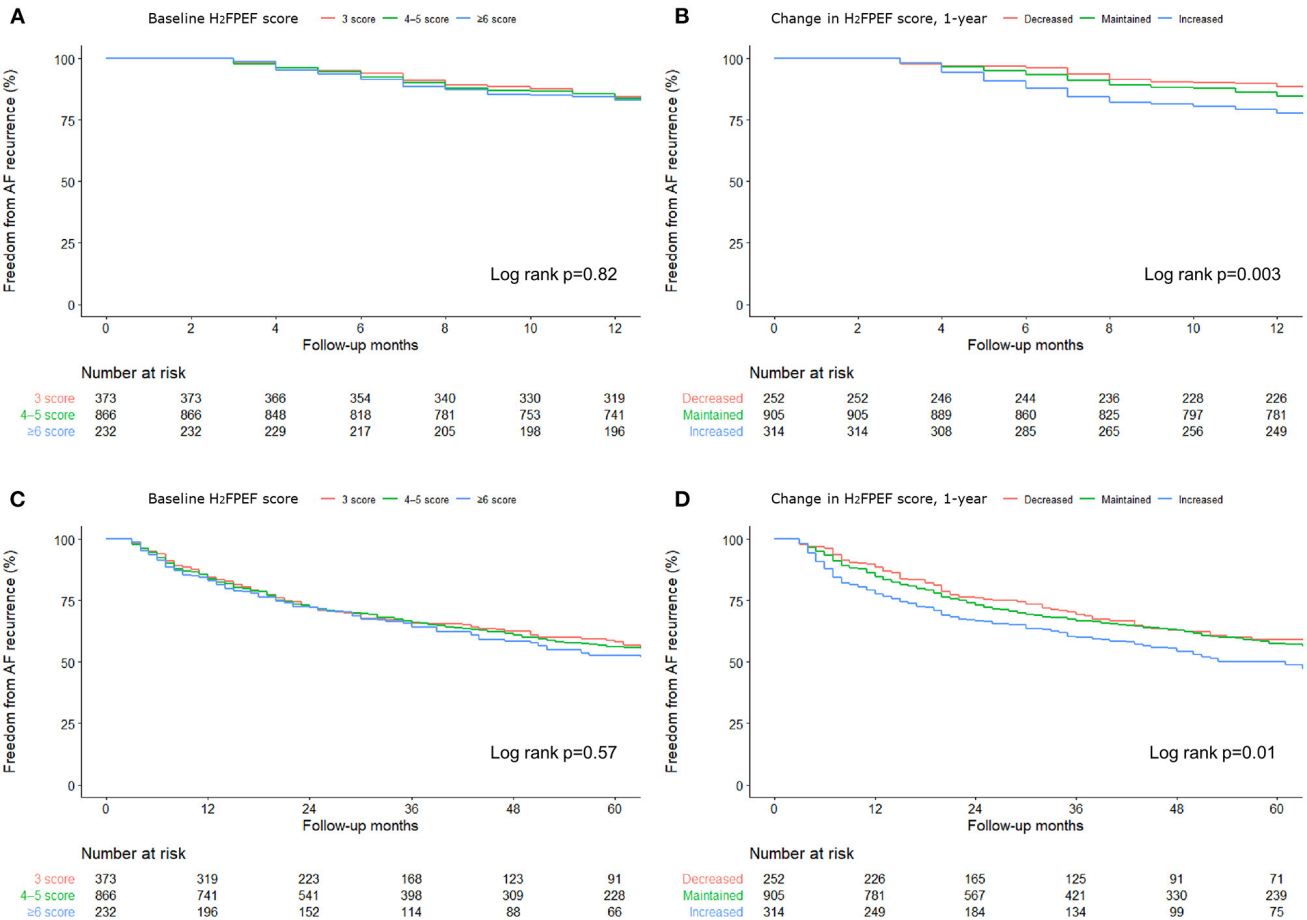
Rate of the freedom from an AF recurrence over 1-year **(A, B)** and during the long-term **(C, D)** after the AFCA based on the baseline H_2_FPEF scores and changes in the 1-year H_2_FPEF scores.

Because we evaluated the H_2_FPEF score before and 1 year after the procedure, we compared the 1-year and long-term clinical recurrence rates of AF separately, depending on the baseline H_2_FPEF scores and ΔH_2_FPEF scores_[1−yr]_. In contrast, the baseline H_2_FPEF scores did not affect the 1-year rhythm outcome (log rank, *p* = 0.82; Figure 3A), and the clinical recurrence of AF was significantly higher in the patients with an increased ΔH_2_FPEF scores_[1−yr]_ (log rank, *p* = 0.003; Figure 3B). In the multivariate Cox regression analysis, increased ΔH_2_FPEF scores_[1−yr]_ [HR, 2.34 (1.36–4.03), *p* = 0.002] and persistent AF [HR, 1.43 (1.01–2.03), *p* = 0.043] were independently associated with an AF recurrence within a year (Supplementary Table 2). During the median follow-up of 56 (32, 87) months, increased ΔH_2_FPEF scores_[1−yr]_ [HR, 1.41 (1.01–1.98), *p* = 0.045], the LA diameter [HR, 1.03 (1.01–1.06), *p* = 0.002], and the LA voltage [HR, 0.59 (0.48–0.73), *p* < 0.001] were independently associated with a long-term AF recurrence (Table 5). The rhythm outcomes in the overall duration were consistent with the 1-year rhythm outcome depending on the baseline H_2_FPEF score (log rank, *p* = 0.57; Figure 3C) or ΔH_2_FPEF score_[1−yr]_ (log rank, *p* = 0.01; Figure 3D). In the subgroup of patients with baseline H_2_FPEF scores of ≥5, the risk of an AF recurrence was significantly higher in the patients with increased ΔH_2_FPEF scores_[1−yr]_ than in those with reduced ΔH_2_FPEF scores_[1−yr]_ (Supplementary Figure 1).

**Table 5:** Cox regression analysis of the predictors of an AF recurrence after the AFCA, during the long-term follow up.

	**Univariable analysis**		**Multivariable analysis**	
	**Unadjusted HR (95% CI)**	***p*-value**	**Adjusted HR (95% CI)**	***p*-value**
Baseline H_2_FPEF scores	1.03 (0.96–1.11)	0.421		
**Baseline H**_**2**_ **FPEF scores**
3 score (reference)	1.00			
4–5 score	1.03 (0.85–1.25)	0.757		
≥6 score	1.14 (0.89–1.46)	0.311		
Change in H_2_FPEF scores, 1 year	1.20 (1.07–1.35)	0.002		
**Change in H**_**2**_ **FPEF scores, 1-year**
Decreased, <0 (reference)	1.00		1.00	
Maintained, 0	1.06 (0.85–1.32)	0.611	1.06 (0.78–1.44)	0.699
Increased, >0	1.38 (1.07–1.78)	0.012	1.41 (1.01–1.98)	0.045
Age	1.00 (1.00–1.01)	0.460		
Female	1.09 (0.92–1.29)	0.307	1.05 (0.82–1.34)	0.694
Persistent AF	1.71 (1.62–2.24)	<0.001	1.19 (0.93–1.53)	0.163
Body mass index	1.03 (1.00–1.06)	0.038		
Smoking	1.04 (0.86–1.20)	0.821		
Alcohol	0.95 (0.81–1.12)	0.547		
CHA_2_DS_2_-VASc score	1.03 (0.98–1.08)	0.221		
Heart failure^*^	1.43 (1.06–1.93)	0.929		
Hypertension	1.14 (0.89–1.46)	0.019		
Diabetes	1.00 (0.80–1.25)	0.973		
Prior stroke/TIA	1.06 (0.84–1.34)	0.640		
Vascular disease	1.05 (0.84–1.31)	0.667		
LA diameter	1.05 (1.04–1.06)	<0.001	1.03 (1.01–1.06)	0.002
LVEF	0.99 (0.97–1.00)	0.024	0.99 (0.97–1.01)	0.203
E/Em	1.00 (0.99–1.02)	0.629		
TR jet velocity	1.01 (0.94–1.09)	0.751		
RVSP	1.02 (1.01–1.03)	<0.001		
LVEDD	1.01 (1.00–1.03)	0.183		
LVESD	1.02 (1.00–1.04)	0.101		
LVMI	1.00 (1.00–1.01)	0.019	1.00 (0.99–1.01)	0.998
eGFR	1.00 (0.99–1.00)	0.380		
hs-CRP	1.00 (0.99–1.01)	0.769		
Pericardial fat volume	1.00 (1.00–1.00)	0.052	1.00 (1.00–1.00)	0.678
LA volume	1.01 (1.01–1.01)	<0.001		
LA mean voltage	0.56 (0.48–0.66)	<0.001	0.59 (0.48–0.73)	<0.001
LA peak pressure	1.01 (1.00–1.02)	0.010	0.99 (0.98–1.01)	0.304

*AF, atrial fibrillation; AFCA, atrial fibrillation catheter ablation; BMI, body mass index; EEm, ratio of the early diastolic mitral inflow velocity (E) to the early diastolic mitral annular velocity (Em); eGFR, estimated glomerular filtration rate; hs-CRP, high sensitivity C-reactive protein; LA, left atrium; LVEDD, left ventricular end diastolic dimension; LVEF, left ventricular ejection fraction; LVESD, left ventricular end systolic dimension; LVMI, left ventricular mass index; RVSP, right ventricular systolic pressure; TIA, transient ischemic attack; TR, tricuspid regurgitation*.

* *Defined as conventional HFpEF diagnosis criteria: left ventricular ejection fraction ≥50% with exertional dyspnea that was not caused by extracardiac causes*.

### Failed Rhythm Control and the ΔH_2_FPEF Score_[1-yr]_

Among the 1,471 patients, 257 (17.5%) had an AF recurrence within a year, and 201 (13.7%) had sinus rhythm restored after using antiarrhythmic drugs, but 56 (3.8%) patients had sustained AF under antiarrhythmic drugs even after cardioversion. The proportion of patients with reduced ΔH_2_FPEF scores_[1−yr]_ tended to be higher without a statistical significance among those with no recurrence (*p* = 0.118, Figure 2C). However, the proportion of patients with increased ΔH_2_FPEF scores_[1−yr]_ was significantly higher in the group with sustained AF after a recurrence than in those with sinus rhythm maintained at a year after the AFCA (*p* = 0.007, Figure 2D).

## Discussion

### Main Findings

In this study, we observed a change in the H_2_FPEF score 1 year after the AFCA in AF patients with a normal LV systolic function. The H_2_FPEF score_[1−yr]_ decreased in 17% of the patients but increased in 21% a year after the AFCA. A high baseline H_2_FPEF score, which is related to LV diastolic dysfunction, was independently associated with a reduced ΔH_2_FPEF score_[1−yr]_. On the other hand, low baseline scores or sustained AF after a recurrence were significantly associated with an increase in the ΔH_2_FPEF scores_[1−yr]_ after the AFCA. Patients with an increased ΔH_2_FPEF score_[1−yr]_ had higher rates of recurrence within a year or longer. Therefore, AFCA improved the H_2_FPEF scores_[1−yr]_ in patients with baseline LV diastolic dysfunction; however, patients with a poor rhythm control and sustained AF despite AFCA had a significant increase in the ΔH_2_FPEF scores_[1−yr]_.

### Atrial Fibrillation and the Ventricular Diastolic Function

AF and LV diastolic dysfunction are closely related and have important features in common, such as age, obesity, hypertension, and diabetes (19). LV diastolic dysfunction has deteriorative effects on the atrial function and structure, which contributes to the development, progression, and maintenance of AF (20). Consequently, AF has an influence on the LV function and LA remodeling (21), and can lead to increasingly sustained AF episodes. Therefore, these two conditions have pathophysiological effects on the occurrence and aggravation of each, and coexistence is associated with a poor prognosis (2, 22, 23). Furthermore, as the AF burden increases chronically, the right ventricular function progressively worsens (24). Reddy et al. (10) proposed a novel risk score, the H_2_FPEF score, which includes all of the abovementioned factors, to diagnose HFpEF patients. We adopted this score to evaluate the prognostic utility in AF patients with a normal LVEF after AFCA.

### Effects of Atrial Fibrillation Catheter Ablation on the Left Venticular Diastolic Function

The data to support the clinical benefits of catheter ablation in symptomatic AF patients with HFrEF are strong and compatible with those from previous clinical trials and meta-analyses (6, 25). However, AF is more potently associated with HFpEF (2) with the prevalence of HFpEF increasing by almost half in AF patients (26). The benefits of AFCA in symptomatic AF patients with HFpEF have been investigated in previous studies (7, 8), but the effects seem less favorable than those in HFrEF patients. Machino-Ohtsuka et al. (7) showed that the longstanding persistent-type AF and a lack of hypertension were factors associated with an improvement in the LV systolic and diastolic indices when sinus rhythm was maintained. Black-Maier et al. (8) showed equivalent rhythm outcomes, all-cause hospitalization, and cardiovascular hospitalization in patients with both HFpEF and HFrEF after AFCA over a median follow-up of 10 months. However, previous studies adopted conventional diagnostic approaches that mainly consisted of symptoms and the LVEF for an HFpEF diagnosis, which show a heterogeneity in terms of the inclusion (9). Thus, we used the H_2_FPEF scores that were newly developed for identifying HFpEF patients and which were superior to the previous diagnostic algorithms. Although the baseline H_2_FPEF scores do not have prognostic value for rhythm outcomes after AFCA, which is consistent with a recent study (27), increased ΔH_2_FPEF scores_[1−yr]_ were independently associated with the rhythm outcomes in this study. Although the cause–result relationship was unclear, sustained AF despite AFCA had a significant correlation with an increased ΔH_2_FPEF score_[1−yr]_.

### Heart Failure With Preserved Ejection Fraction, a Good Candidate for Atrial Fibrillation Catheter Ablation

Among the patients with a normal LV systolic function, which subgroup is the most helpful based on the H_2_FPEF scores after AFCA? Those with the most significant decrease in the ΔH_2_FPEF scores_[1−yr]_ were those with a baseline HFpEF score of ≥6, i.e., successful rhythm control by AFCA can significantly improve the LV diastolic function in patients with HFpEF. Furthermore, the absence of diabetes, a higher LVEF, and a lower pericardial fat volume were associated with decreased ΔH_2_FPEF scores_[1−yr]_, which suggested that metabolic factors may have influenced the recovery of the LV diastolic function.

### Limitations

This study had several limitations. First, since the current study was conducted in a single center and included a relatively small number of patients, the findings cannot be generalized to all patients with a normal LV systolic function. However, there was also an advantage of this single-center cohort in that the ablation and rhythm follow-up protocols were consistent. Second, although we performed a regular rhythm follow-up in all included patients, the exact AF burden could not be assessed by the Holter monitoring. Third, we excluded patients who did not have both baseline and 1-year follow-up echocardiograms with all parameters taken from the same institute, to calculate appropriate H_2_FPEF scores. Fourth, there was some discrepancy between the HF clinically judged by the CH_2_A_2_DS-VASc and H_2_FPEF scores because the clinical HF was classified mainly by the LV systolic function. Finally, because of the limited follow-up duration, we could not determine the long-term changes in the biventricular function and other clinical outcomes in this study. Future prospective and controlled studies are warranted.

## Conclusion

The H_2_FPEF scores decreased in 17% and increased in 21% of the patients with a normal LV function at 1 year after the AFCA. AFCA has shown a tendency to improve the H_2_FPEF scores_[1−yr]_ in the patients with an abnormal diastolic function. However, a poor rhythm control and sustained AF after the AFCA were significantly associated with an increase in the ΔH_2_FPEF score_[1−yr]_. An increased ΔH_2_FPEF score_[1−yr]_ was an independent prognostic factor for poorer rhythm outcomes after the AFCA.

## Data Availability Statement

Data supporting the findings of this study are available from the corresponding author upon reasonable request.

## Ethics Statement

The studies involving human participants were reviewed and approved by The institutional review board of the Yonsei University Health system. The patients/participants provided their written informed consent to participate in this study.

## Author Contributions

HN-P and MK designed the study, analyzed and interpreted the data, drafted the manuscript, and did the final approval of the manuscript submission. HTY, T-HK, J-SU, BYJ, and M-HL interpreted data and contributed to acquiring patients' clinical data. All authors contributed to the article and approved the submitted version.

## Conflict of Interest

The authors declare that the research was conducted in the absence of any commercial or financial relationships that could be construed as a potential conflict of interest.

## Publisher's Note

All claims expressed in this article are solely those of the authors and do not necessarily represent those of their affiliated organizations, or those of the publisher, the editors and the reviewers. Any product that may be evaluated in this article, or claim that may be made by its manufacturer, is not guaranteed or endorsed by the publisher.

## Abbreviations

ΔH2FPEF score_[1−yr]_: 1-year change in the H2FPEF score
SustainAF_[1−yr]_: sustained AF after a recurrence within a year.
