# Supplementary material for: One-Year Change in the H2FPEF Score After Catheter Ablation of Atrial Fibrillation in Patients With a Normal Left Ventricular Systolic Function
Source: Front Cardiovasc Med. 2021 Aug 3;8:699364. doi: 10.3389/fcvm.2021.699364 (PMC8369029; doi:10.3389/fcvm.2021.699364)

Supplementary Table 1. Logistic regression analysis for the predictors of the baseline high H_2FPEF scores (≥6)

	Univariable analysis		Multivariable analysis	
	**Unadjusted HR (95% CI)**	**p value**	**Adjusted HR (95% CI)**	**p value**
Age	1.11 (1.09-1.13)	<0.001		
Female	2.13 (1.60-2.83)	<0.001	2.31 (1.35-3.93)	0.002
Paroxysmal AF	0.74 (0.55-0.99)	0.042	1.06 (0.66-1.70)	0.812
BMI	1.14 (1.09-1.20)	<0.001		
Smoking	0.64 (0.46-0.87)	0.004	0.96 (0.53-1.75)	0.902
Alcohol	0.54 (0.40-0.72)	<0.001	0.79 (0.46-1.38)	0.412
CHA_2DS_2-VASc score	1.89 (1.71-2.07)	<0.001		
Heart failure*	2.79 (1.78-4.38)	0.001		
Hypertension	13.9 (8.84-21.9)	<0.001		
Diabetes	2.39 (1.71-3.34)	<0.001	1.45 (0.87-2.41)	0.155
Prior stroke/TIA	1.76 (1.21-2.57)	0.003	1.41 (0.83-2.42)	0.206
Vascular disease	2.02 (1.41-2.90)	<0.001	1.25 (0.71-2.20)	0.435
LA diameter	1.11 (1.08-1.14)	<0.001	1.09 (1.04-1.13)	<0.001
LVEF	1.02 (0.99-1.04)	0.172		
E/Em	1.28 (1.23-1.33)	<0.001		
TR jet velocity	6.63 (4.21-10.5)	<0.001		
RVSP	1.16 (1.13-1.18)	<0.001		
LVEDD	1.02 (0.98-1.05)	0.299		
LVESD	1.00 (0.96-1.03)	0.825		
LVMI	1.03 (1.02-1.03)	<0.001	1.02 (1.01-1.03)	0.002
eGFR	0.97 (0.96-0.98)	<0.001	0.98 (0.97-0.99)	<0.001
hs-CRP	1.03 (1.01-1.05)	0.007	1.00 (0.97-1.03)	0.974
Pericardial fat volume	1.01 (1.00-1.01)	<0.001	1.01 (1.00-1.01)	0.015
LA volume	1.01 (1.01-1.01)	<0.001		
LA mean voltage	0.54 (0.41-0.72)	<0.001	0.76 (0.52-1.10)	0.144
LA peak pressure	1.03 (1.02-1.05)	<0.001	1.01 (0.99-1.03)	0.392

Abbreviation: AF, atrial fibrillation; AFCA, atrial fibrillation catheter ablation; BMI, body mass index; EEm, ratio of the early diastolic mitral inflow velocity (E) to the early diastolic mitral annular velocity (Em); eGFR, estimated glomerular filtration rate; hs-CRP, high sensitivity C-reactive protein; LA, left atrium; LVEDD, left ventricular end diastolic dimension; LVEF, left ventricular ejection fraction; LVESD, left ventricular end systolic dimension; LVMI, left ventricular mass index; RVSP, right ventricular systolic pressure; TIA, transient ischemic attack; TR, tricuspid regurgitation

* Defined as conventional HFpEF diagnosis criteria: left ventricular ejection fraction ≥50% with exertional dyspnea that was not caused by extracardiac causes.

Supplementary Table 2. Cox regression analysis of the predictors of an AF recurrence after the AFCA, at 1-year

	Univariable analysis		Multivariable analysis	
	**Unadjusted HR (95% CI)**	**p value**	**Adjusted HR (95% CI)**	**p value**
Baseline H_2FPEF scores	1.00 (0.89-1.12)	0.991		
Baseline H_2FPEF scores				
3 score (reference)	1.00			
4-5 score	1.06 (0.79-1.43)	0.681		
≥6 score	1.13 (0.77-1.67)	0.526		
Change in H_2FPEF scores, 1-year	1.39 (1.16-1.65)	<0.001		
Change in H_2FPEF scores, 1-year				
Decreased, <0 (reference)	1.00		1.00	
Maintained, 0	1.30 (0.89-1.90)	0.170	1.65 (1.00-2.74)	0.052
Increased, >0	1.92 (1.27-2.89)	0.002	2.34 (1.36-4.03)	0.002
Age	1.00 (0.99-1.01)	0.755		
Female	0.91 (0.69-1.19)	0.501	0.91 (0.63-1.30)	0.591
Persistent AF	1.80 (1.41-2.30)	<0.001	1.43 (1.01-2.03)	0.043
Body mass index	1.03 (0.99-1.07)	0.188		
Smoking	1.04 (0.81-1.34)	0.768		
Alcohol	0.91 (0.71-1.17)	0.468		
CHA_2DS_2-VASc score	1.02 (0.94-1.10)	0.702		
Heart failure*	1.02 (0.63-1.67)	0.929		
Hypertension	1.14 (0.89-1.46)	0.292		
Diabetes	1.15 (0.83-1.60)	0.393		
Prior stroke/TIA	1.06 (0.73-1.52)	0.768		
Vascular disease	1.10 (0.78-1.57)	0.580		
LA diameter	1.05 (1.02-1.07)	<0.001	1.02 (0.99-1.05)	0.186
LVEF	0.99 (0.97-1.01)	0.369		
E/Em	0.99 (0.96-1.02)	0.577		
TR jet velocity	0.98 (0.84-1.16)	0.840		
RVSP	1.01 (1.00-1.03)	0.147		
LVEDD	1.02 (0.99-1.05)	0.183		
LVESD	1.02 (0.99-1.05)	0.103		
LVMI	1.01 (1.00-1.01)	0.019	1.01 (1.00-1.01)	0.135
eGFR	1.00 (0.99-1.01)	0.596		
hs-CRP	1.00 (0.97-1.02)	0.723		
Pericardial fat volume	1.00 (1.00-1.00)	0.067	1.00 (1.00-1.00)	0.963
LA volume	1.00 (1.00-1.01)	0.002		
LA mean voltage	0.67 (0.53-0.85)	0.001	0.77 (0.58-1.02)	0.067
LA peak pressure	1.01 (0.99-1.02)	0.376		

Abbreviation: AF, atrial fibrillation; AFCA, atrial fibrillation catheter ablation; BMI, body mass index; EEm, ratio of the early diastolic mitral inflow velocity (E) to the early diastolic mitral annular velocity (Em); eGFR, estimated glomerular filtration rate; hs-CRP, high sensitivity C-reactive protein; LA, left atrium; LVEDD, left ventricular end diastolic dimension; LVEF, left ventricular ejection fraction; LVESD, left ventricular end systolic dimension; LVMI, left ventricular mass index; RVSP, right ventricular systolic pressure; TIA, transient ischemic attack; TR, tricuspid regurgitation

* Defined as conventional HFpEF diagnosis criteria: left ventricular ejection fraction ≥50% with exertional dyspnea that was not caused by extracardiac causes

Supplementary Figure 1. Risk of an AF recurrence according to the change in the 1-year H_2FPEF scores after the AFCA based on the individual baseline H_2FPEF scores.

Abbreviations: AF, atrial fibrillation; AFCA, atrial fibrillation catheter ablation; LA, left atrium; LVEF, left ventricular ejection fraction; LVMI, left ventricular mass index

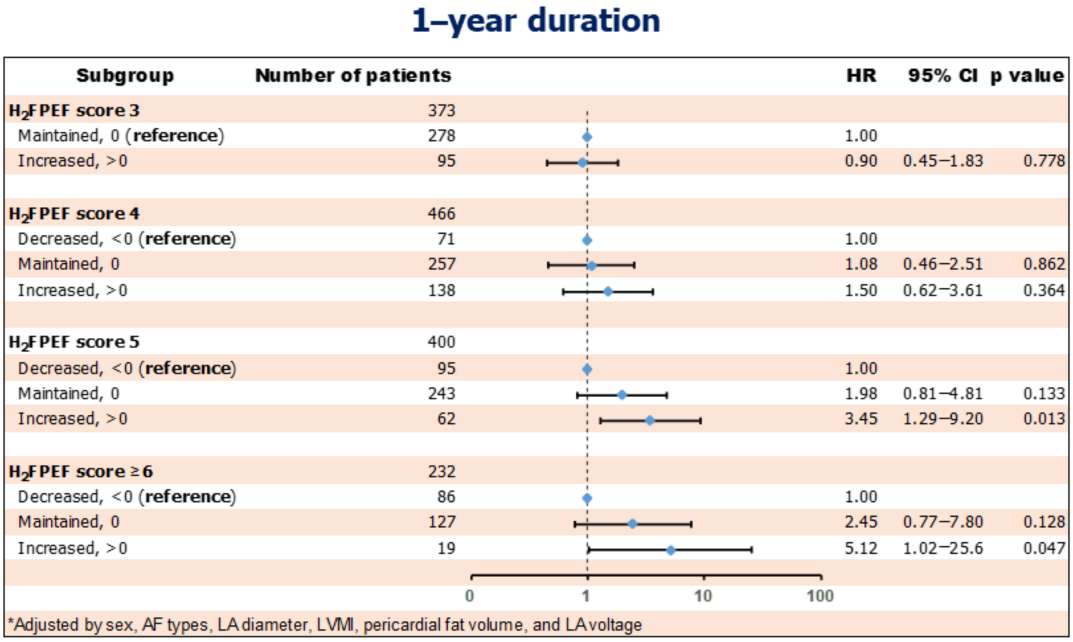

Supplement: Supplementary file 1 [file Data_Sheet_1.pdf]
